# The updated duplex fluorescence quantitative RT-PCR assay for simultaneous detection of PRRSV-1 and PRRSV-2

**DOI:** 10.3389/fcimb.2025.1616898

**Published:** 2025-06-19

**Authors:** Xiaoxiao Tian, Haojie Wang, Zeqing Liu, Ziyi Wei, Yongbo Yang, Haiwei Wang, Guoqing Liu, Hao Song, Xinyi Huang, Tongqing An

**Affiliations:** ^1^ State Key Laboratory for Animal Disease Control and Prevention, Harbin Veterinary Research Institute, Chinese Academy of Agricultural Sciences, Harbin, China; ^2^ Heilongjiang Provincial Key Laboratory of Veterinary Immunology, Harbin, China

**Keywords:** PRRSV-1, PRRSV-2, duplex fluorescence quantitative RT-PCR, swine, detection

## Abstract

**Introduction:**

Porcine reproductive and respiratory syndrome (PRRS) is one of the most economically devastating infectious diseases in the global swine industry. With the continuous mutation and recombination of PRRSV, existing detection methods frequently result in false negatives, further complicating the prevention and control of PRRS.

**Methods:**

The duplex real-time quantitative RT-PCR (RT-qPCR) for the simultaneous detection of PRRSV-1 and PRRSV-2 was developed by designing specific primers and probes based on the ORF6 gene, which is different from conventional nucleic acid detection methods that are typically based on the ORF7 gene.

**Results:**

The method showed high specificity for exclusively detecting PRRSV-1 and PRRSV-2, with no cross-reactivity observed against other porcine pathogens. The limit of detection (LOD) was 8.42 copies for PRRSV-1 and 7.84 copies for PRRSV-2. Intra-assay coefficients of variation (CVs) were 0.22–1.07% and inter-assay CVs were 0.52–1.28%. A total of 356 clinical samples were detected using the developed duplex RT-qPCR and compared to the WOAH-recommended RT-qPCR assay and commercial universal PRRSV RT-qPCR detection kit. The assay established in this study demonstrated higher positivity rates, indicating its superior sensitivity.

**Discussion:**

An efficient, sensitive, and accurate method for the detection and differentiation of PRRSV-1 and PRRSV-2 was developed and applied to the detection and monitoring of PRRSV.

## Introduction

Porcine reproductive and respiratory syndrome (PRRS) is an economically infectious disease that causes reproductive failure in sows and severe respiratory disorders in pigs of all ages (Benfield et al., 1992; Han et al., 2014). PRRS virus (PRRSV) is an enveloped RNA virus of the genus *Betaarterivirus*, family *Arteriviridae*, and order *Nidovirales* (Meulenberg, 2000). PRRSVs can be categorized into two distinct species, *Betaarterivirus suid 1* (PRRSV-1, previously known as European type) and *Betaarterivirus suid 2* (PRRSV-2, previously known as North American type), which exhibit 60% nucleotide identity at the whole-genome level (Brinton et al., 2021). Based on the newly lineage classification in 2023, PRRSV-2 is divided into eleven lineages (L1-L11), and the main epidemic lineages in China are L1, L3, L5, and L8 (Tian et al., 2025; Yim-Im et al., 2023). PRRSV was first isolated in China in 1996. In 2006, highly pathogenic PRRSV (HP-PRRSV), characterized by high fever, high morbidity, and high mortality, emerged in Jiangxi province (Tian et al., 2007). The variant rapidly spread throughout China and gradually replaced the classical strain, becoming the dominant PRRSV strain (Zhou and Yang, 2010; Guo et al., 2018). In 2012, a new subgroup of PRRSV strains named NADC30-like (NL-PRRSV) was identified in China, and has become the most prevalent strain in China since 2016 (Zhou et al., 2015). All NL-PRRSV strains have the same amino acids deletion in NSP2 protein (111 + 1 + 19) of NADC30 strain isolated in the United States in 2008, which are considered to be imported from North American and adapted in China (Brockmeier et al., 2012). In 2017, NADC34-like was first reported in China, and have gradually become the predominant epidemic strains (Zhang et al., 2018; Xu et al., 2022). Although PRRSV-2 is the mainstream strain in China, the detection rate of PRRSV-1 in China has been increasing in recent years (Chen et al., 2011). For example, in 750 samples collected from 50 breeding farms in Guangdong, the positive rate of PRRSV-1 was as high as 24.8% (Zhai et al., 2018). To date, PRRSV-1 has been detected in at least 23 regions in China (Sun et al., 2023). Due to the high variability and recombination rate, PRRSV exist complex genetic diversity, which increases the difficulty of PRRSV prevention and control.

Viral isolation is the standard method for diagnosis of PRRS, but it is consuming and complicated. Rapid and reliable detection method is of great significance for the timely diagnosis of PRRS. At present, PRRSV detection methods are mainly for nucleic acids, antibodies and antigens. For example, indirect immunofluorescence assay (Wang et al., 2017), enzyme-linked immunosorbent assay (Kittawornrat et al., 2012; Xiao et al., 2014), polymerase chain reaction (PCR) (Peng et al., 2017), reverse transcription quantitative PCR (RT-qPCR) (Chen et al., 2019; Zhang et al., 2022), reverse transcription loop-mediated isothermal amplification (Zhang et al., 2011) and reverse transcription recombinase polymerase amplification have been constructed (Tian et al., 2022). Among these methods, RT-qPCR has the advantages such as low cost, rapid detection, and high sensitivity, and is currently the most widely used technique for PRRSV detection. However, existing RT-qPCR assays for PRRSV are primarily designed for the detection of PRRSV-2 strains prevalent in China. With the ongoing genetic evolution of PRRSV-2, these assays are increasingly prone to false negatives. Furthermore, given the rising detection rate of PRRSV-1 in China, there is also an urgent need to update quantitative detection methods for PRRSV-1. Therefore, regular updates of PRRSV RT-qPCR primers and probes are vital to ensure the accuracy of clinical diagnostics.

In this study, we downloaded all the PRRSV-1 (n=74) and PRRSV-2 (n=512) complete genomes in China from GenBank by June 2024, and established a duplex RT-qPCR for PRRSV-1 and PRRSV-2 based on conserved regions. This method aims to provide an efficient and reliable molecular diagnostic tool for the detection and monitoring of PRRSV.

## Materials and methods

### Bacteria and viruses

Classic PRRSV (C-PRRSV) CH-1a strain (GenBank accession no. AY032626.1), NL-PRRSV HeB-108 strain (GenBank accession no. MN046224.1), HP-PRRSV HuN4 strain (GenBank accession no. EF635006.1), PRRSV-1, Pseudorabies virus (PRV), Porcine epidemic diarrhea virus (PEDV), Classical swine fever virus (CSFV), Porcine circovirus 2 (PCV2), *Streptococcus suis* (*S. suis*, SS), and *Gracilaria parapsilosis* (*G. parapsilosis*, GPS) were preserved in our laboratory.

### Collection of the clinical samples

A total of 356 samples, including lungs, spleens, kidneys, intestines, lymph nodes, livers, hearts, throat swabs, and blood, were collected from various farms across Guangdong, Hebei, Shandong, Zhejiang, Heilongjiang, and Liaoning provinces of China between 2019 and 2024. These samples primarily originated from sows exhibiting reproductive disorders and pigs suspected of PRRSV infection.

### Design of primers and probes

**Figure 1:**
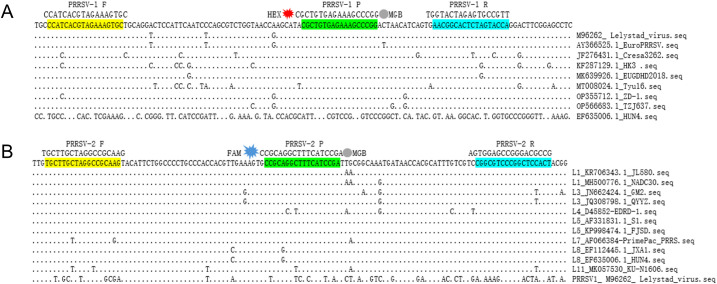
Positions of the duplex RT-qPCR primers and probes in the aligned ORF6 gene. **(A)** The position of primer and probe of PRRSV-1 used in this study. **(B)** The position of primer and probe of PRRSV-2 used in this study; due to the absence of ORF6 gene sequences for lineages L2, L6, L9, and L10 in GenBank, these lineages could not be included in the conservation analysis. The GenBank accession numbers and corresponding strain names of PRRSV isolates are detailed on the right. Nucleotide residues that match the majority are indicated by dots.

All complete genomic sequences of PRRSV-1 (n=74) and PRRSV-2 (n=512) in China were downloaded from GenBank database by June 2024, and respectively aligned using DNASTAR (DNASTAR Inc., Madison, WI, USA) to identify the most conserved region (**Figure 1**). Based on the conserved regions within the ORF6 gene, primers and probes were designed using Primer Premier 5 software (Premier Biosoft International, Palo Alto, CA, USA) to optimize melting temperature, GC content, and minimize secondary structures. The selected primers and probes were then evaluated for specificity before being synthesized by Comate Bioscience Co., Ltd. (Jilin, China) at a working concentration of 10 μM. The primer and probe sequences in this experiment are listed in **Table 1**.

**Table 1:** Primers and probes used in this study.

Pathogens	Sequence (5’-3’)	Product size (bp)
PRRSV-1	PRRSV-1 F: CCATCACGTAGAAAGTGC PRRSV-1 R: TGGTACTAGAGTGCCGTT PRRSV-1 Probe: HEX- CGCTGTGAGAAAGCCCGG-MGB	109
PRRSV-2	PRRSV-2 F: TGCTTGCTAGGCCGCAAG PRRSV-2 R: AGTGGAGCCGGGACGCCG PRRSV-2 Probe: FAM- CCGCAGGCTTTCATCCGA -MGB	120
LV4.2.1 (AY588319)	F1: ATGGGAGGCCTAGACGAT	900
	R1: AATTAACTTGCACCCTGA	
HeB-108 (MN046224)	F2: CGGAGTACAAACAAGGTC	398
	R2: CCAGCATCTGGCACAGCTGA	

### Nucleic acid extraction

Nucleic acid of clinical samples or cell cultures was extracted following the instruction of BayBiopure Magnetic Bead-based Nucleic Acid Extraction Kit. Briefly, tissues were mixed with 500 μL of PBS and homogenized. Nasal swabs were added 500 μL PBS and mixed well. Subsequently, tissue homogenates and swab samples were centrifuged at 8000 rpm for 2 min, and the supernatant was collected for nucleic acid extraction. To obtain the DNA/RNA from either clinical samples or infected culture fluids, add 200 μL of supernatant and 20 μL of proteinase K into a pre-aliquoted deep-well plate, and the mixture was then placed into a nucleic acid purification system for extraction. Finally, the extracted nucleic acid was eluted with 50 μL of nuclease-free water.

### Construction of the standard plasmids

The construction of the standard recombinant plasmids was performed as described previous with minor modifications (Liu et al., 2022). Briefly, the targeted fragments of ORF6 gene were amplified via PCR from the cDNA of PRRSV-1 (LV4.2.1 strain) and PRRSV-2 (HeB-108 strain), respectively (**Table 1**). The PCR products were purified and cloned into the pMD18-T vector (TaKaRa, Dalian, China), and subsequently transformed into E. coli DH5α cells (TaKaRa, Dalian, China). The recombinant standard plasmids were confirmed via sequencing, and named pMD-PRRSV-1 and pMD-PRRSV-2, respectively. The concentration of the standard plasmid was determined using a NanoDrop spectrophotometer (Thermo Fisher, Waltham, MA, USA), and the copy number was calculated using the following formula:

Plasmid (copies/μL)= (6.02 × 10^23^)×(X ng/μL × 10^−9^)/plasmid length(bp)×660.

### Optimization of the reaction parameters

RT-qPCR experiment was performed using One-Step PrimeScript™ RT-PCR Kit (TaKaRa, Dalian, China) according to the manufacturer’s instruction. To determine the optimal reaction conditions, the duplex RT-qPCR experiments with different annealing temperatures (56°C, 57.5°C, 59°C, 60°C, 61.5°C and 63°C), concentrations of each primer and probe (0.1, 0.2, 0.3, 0.4, 0.5, and 0.6 μM), and number of cycles (30, 35, 40, 45, 50) were performed using the mixture of two standard plasmids of different concentrations (10^8^, 10^6^, 10^4^, 10^2^ copies/µL) as a template. Optimal reaction conditions were selected based on the cycle threshold (Ct) values and amplification efficiency.

### Generation of the standard curves

The mixture of two standard plasmids (at a ratio of 1:1) with concentrations ranging from 2×10^8^ to 2×10^1^ copies/μL (final reaction concentrations: 1×10^8^ to 1×10^1^ copies/μL) was used as a template for amplification to generate the standard curves. To ensure result reliability, all samples were tested in triplicate, and the entire experiment was independently repeated three times.

### Specificity analysis

To evaluate the specificity of the duplex RT-qPCR, nucleic acids from PRRSV-1, three PRRSV-2 subtypes (including C-PRRSV, HP-PRRSV, and NL-PRRSV), and other swine pathogens (including PRV, PPV1, PCV2, PEDV, CSFV, SS, and GPS) were used as templates. Plasmid standards pMD-PRRSV-1 and pMD-PRRSV-2 were used as positive controls, and distilled water was used as the negative control. To ensure the reliability of the results, each sample was tested in triplicate, and the entire experiment was independently repeated three times.

### Sensitivity analysis

To evaluate the sensitivity of the duplex RT-qPCR, the mixture of two standard plasmids (at a ratio of 1:1) with concentrations ranging from 2×10^8^ to 2×10^1^ copies/μL (final reaction concentrations: 1×10^8^ to 1×10^1^ copies/μL) was used as a template for amplification. The limit of detection (LOD) of the method was determined based on Ct values obtained from templates with different concentrations and analyzed using PROBIT regression in SPSS software (Zhang et al., 2022). Each concentration was tested in triplicate, and the entire experiment was independently repeated three times to ensure reliability.

### Repeatability analysis

To evaluate the repeatability of the duplex RT-qPCR, the mixture of two standard plasmids (at a ratio of 1:1) with concentrations of 2×10^8^, 2×10^6^, 2×10^4^, and 2×10^2^ copies/μL (final reaction concentrations: 1×10^8^, 1×10^6^, 1×10^4^, and 1×10^2^) was used as a template for amplification. The intra-assay coefficients of variation (CVs) was assessed by amplifying in triplicate one day, and the inter-assay CVs was assessed by amplifying in three different times, with an interval of 1 week.

### Detection of PRRSV-1 and PRRSV-2 in clinical samples

A total of 356 clinical samples collected from different pig farms in China were analyzed in this study. RNA was extracted from the clinical samples, and detected using the RT-qPCR method established in this study. For validation, the samples were concurrently tested using the WOAH-recommended real-time RT-PCR (WOAH Terrestrial Manual 2021-Chapter 3.9.6) and commercial universal PRRSV RT-qPCR detection kit to assess the accuracy of the method established in this study.

## Results

### Construction of the standard plasmids

The ORF6 gene of PRRSV-1 and PRRSV-2 were amplified, respectively, and used to the recombinant standard plasmids. The plasmids were confirmed by Sanger sequencing, and named pMD-PRRSV-1 and pMD-PRRSV-2, respectively. The original concentrations of the plasmids were determined to be 4.5×10^10^ and 5.3×10^10^ copies/μL, respectively. The final concentrations of both were adjusted to 2×10^8^ copies/μL.

### Optimization of the reaction parameters

**Figure 2:**
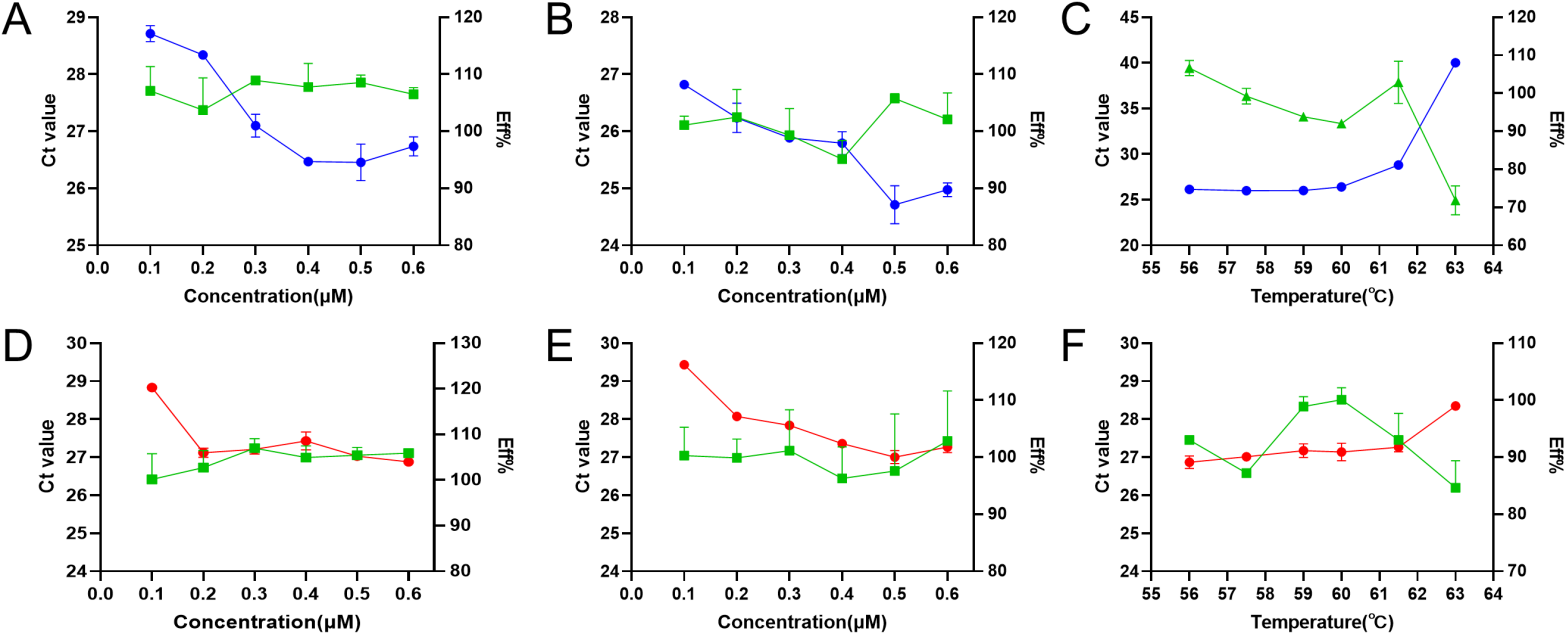
Optimization of annealing temperature, primer concentration, and probe concentration for duplex RT-qPCR. The optimization of primer concentration **(A)**, probe concentration **(B)**, and annealing temperature **(C)** for PRRSV-1, and the optimization of primer concentration **(D)**, probe concentration **(E)**, and annealing temperature **(F)** for PRRSV-2. The blue, red, and green lines represent the Ct values of PRRSV-1, PRRSV-2, and the amplification efficiency, respectively.

After optimization of the reaction parameters of annealing temperatures and the concentration of primers and probes, the optimal parameters of the duplex RT-qPCR were obtained (**Figure 2**). The optimal reaction system in a total volume of 20 μL including 2×One Step RT-PCR Buffer III 10 μL, TaKaRa Ex Taq HS 0.4 μL, PrimeScript RT Enzyme Mix II 0.4 μL, PRRSV-1-F 1 μL, PRRSV-1-R 1 μL, PRRSV-1-Probe 1 μL, PRRSV-2-F 0.4 μL, PRRSV-2-R 0.4 μL, PRRSV-2-Probe 1 μL, RNA 2 μL, ddH_2_O 2.4 μL. Cycle number optimization showed that 30 and 35 cycles resulted in insufficient amplification, whereas 45 and 50 cycles led to elevated background signals and non-specific amplification. Therefore, 40 cycles were selected as optimal. The one-step amplification program was as follows: 42°C for 5 min, 95°C for 10 s, and then 40 cycles of 95°C for 5 s, 59°C for 25 s. Based on these optimized conditions, the established duplex RT-qPCR was evaluated for its specificity, sensitivity, and reproducibility.

### Generation of the standard curves

**Figure 3:**
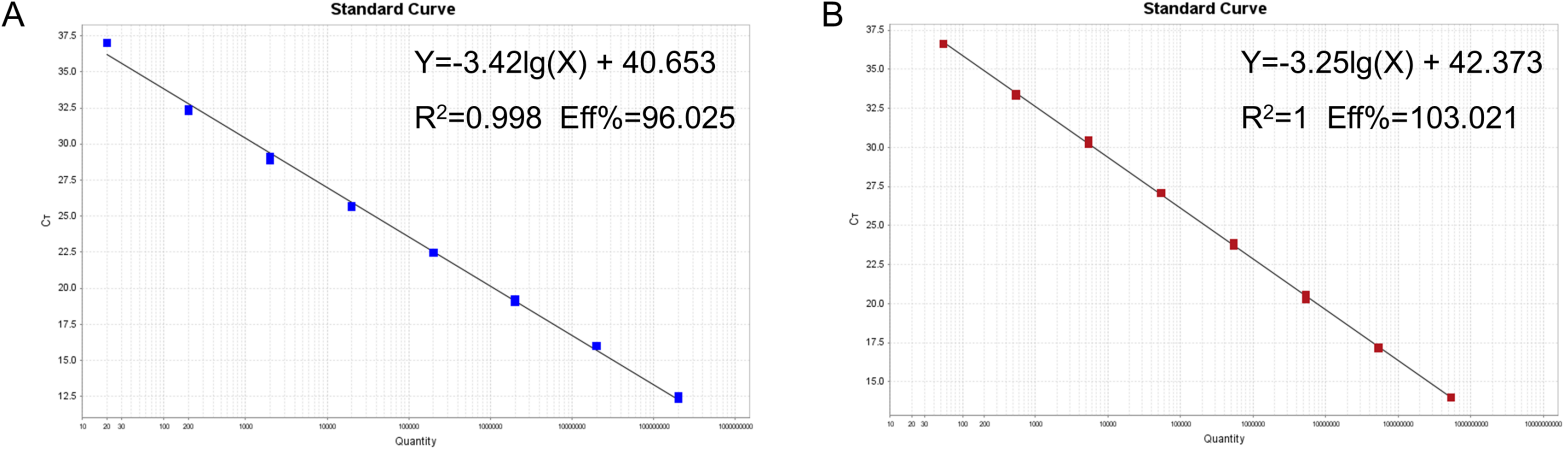
Establishment of the standard curves for recombinant plasmids from targeting virus strains. Standard curves for the **(A)** PRRSV-1 and **(B)** PRRSV-2.

The mixture of two standard plasmids (at a ratio of 1:1) with concentrations ranging from 2×10^8^ to 2×10^1^ copies/μL (final reaction concentrations: 1×10^8^ to 1×10^1^ copies/μL) was used as a template for amplification to generate the standard curves. The results showed that PRRSV-1 (slope = −3.421, R^2^ = 0.998, Eff% = 96.025) and PRRSV-2 (slope = −3.252, R^2^ = 1, Eff% =103.021) had good correlation coefficients (R^2^≥0.998) and amplification efficiencies (**Figure 3**).

### Specificity analysis

**Figure 4:**
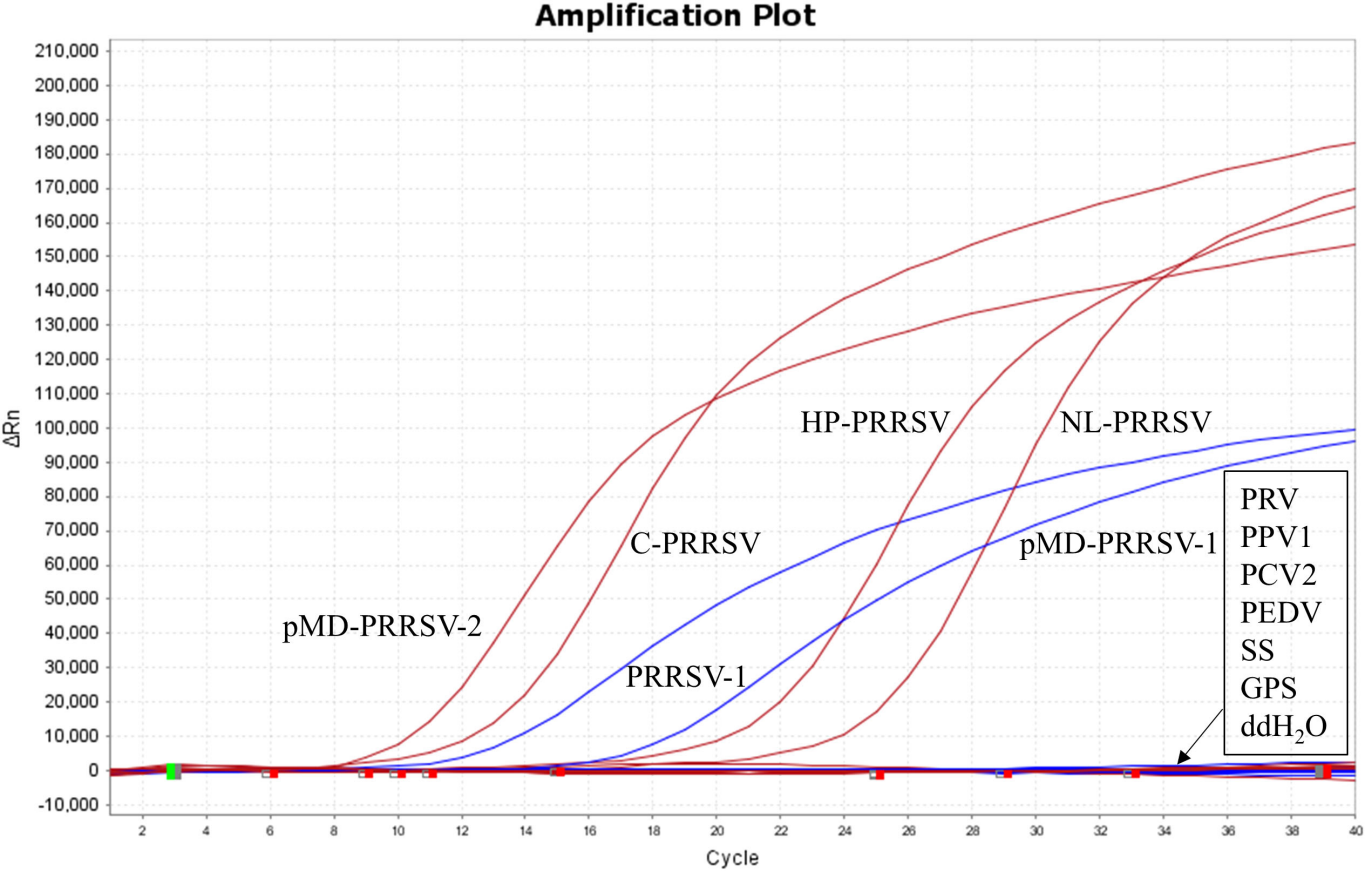
Specificity test results against various swine pathogens. Nucleic acid extracts from PRRSV-1, three PRRSV-2 subtypes (including C-PRRSV, HP-PRRSV, and NL-PRRSV), and other swine pathogens (including PRV, PPV1, PCV2, PEDV, CSFV, SS, and GPS) were used as templates.

The nucleic acids of PRRSV-1, three PRRSV-2 subtypes (including C-PRRSV, HP-PRRSV, and NL-PRRSV), and other swine pathogens (including PRV, PPV1, PCV2, PEDV, CSFV, SS, and GPS) were used to evaluate the specificity of the developed duplex RT-qPCR. The results showed that the assay could detect PRRSV-1 and PRRSV-2 without cross-reactivity to swine pathogens, indicating good specificity of the assay (**Figure 4**).

### Sensitivity analysis

**Figure 5:**
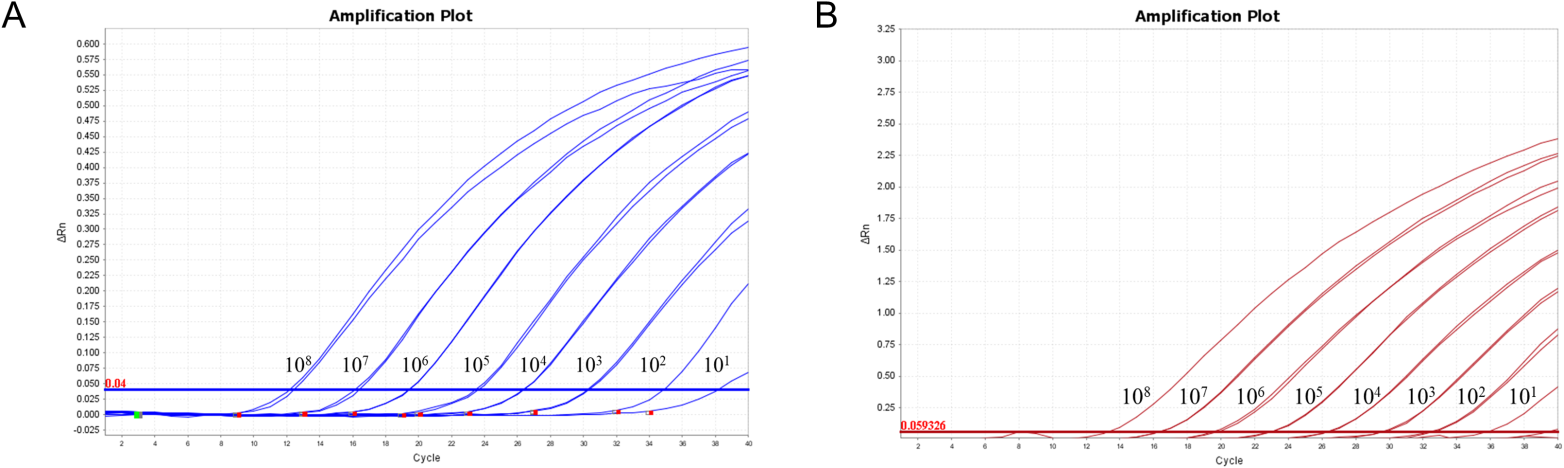
Sensitivity of the duplex RT-qPCR established in this study. The figures show the amplification curves of the standard plasmids pMD-PRRSV-1 and pMD-PRRSV-2 with different concentrations. The final concentrations of the plasmids ranged from 1×10^8^-1×10^1^ copies/μL.

**Figure 6:**
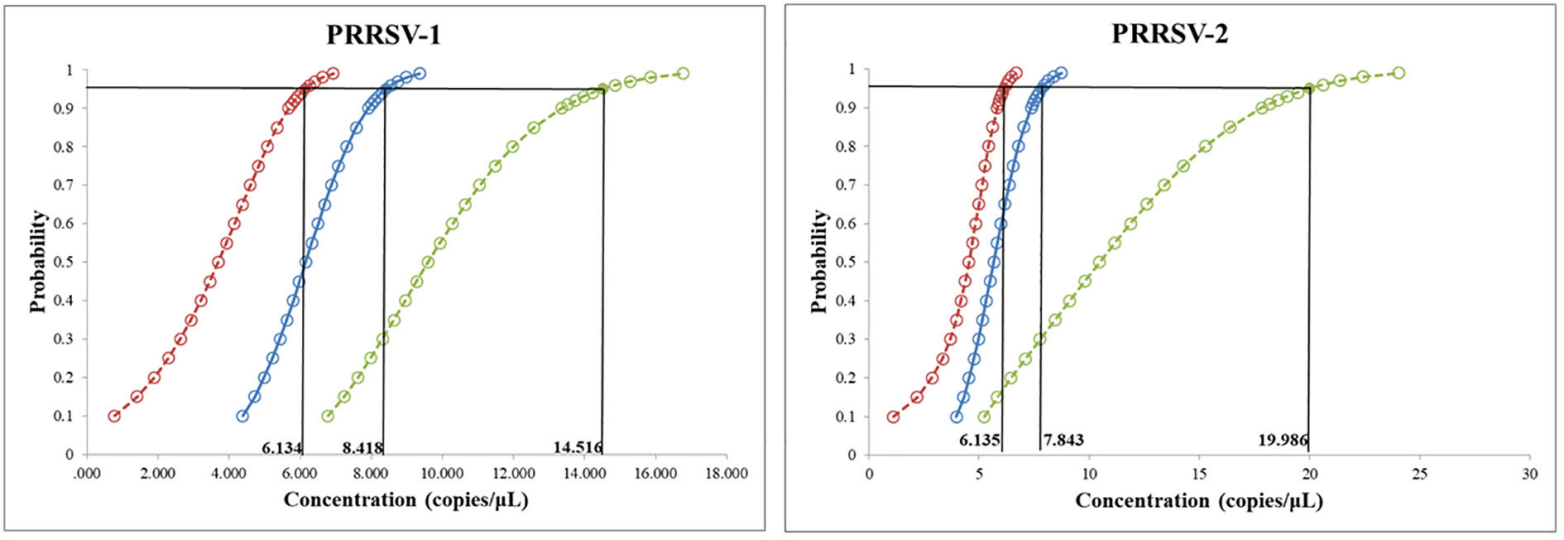
PROBIT analysis of the detection limit of the duplex RT-qPCR stablished in this study. The LODs of pMD-PRRSV-1 and pMD-PRRSV-2 were determined to be 8.42 copies (95% confidence interval: 6.134–14.516) and 7.84 copies (95% confidence interval: 6.135–19.986), respectively.

The mixture of two standard plasmids (at a ratio of 1:1) with concentrations ranging from 2×10^8^ to 2×10^1^ copies/μL (final reaction concentrations: 1×10^8^ to 1×10^1^ copies/μL) was used as a template for amplification to evaluate the sensitivity of the assay. The results showed that the LOD of PRRSV-1 and PRRSV-2 was 1×10^1^ copies/μL (**Figure 5**). Subsequently, recombinant plasmid standards with concentrations of 80, 40, 20, 10, 5, and 2.5 copies/μL were prepared, with 25 replicates for each concentration. The detection was carried out using the method established in this study. As shown in **Table 2**, the average Ct values and detection rates for plasmid standards pMD-PRRSV-1 and pMD-PRRSV-2 at each gradient were recorded. PROBIT regression analysis using SPSS software determined the detection limits of pMD-PRRSV-1 and pMD-PRRSV-2 to be 8.42 copies (95% confidence interval: 6.134–14.516) and 7.84 copies (95% confidence interval: 6.135–19.986), respectively (**Figure 6**).

**Table 2:** Average Ct values and detection rates of plasmid standards at different gradients.

Recombinant plasmid	Concentrations (copies/μL)	Samples	Duplex RT-qPCR	
			Ct (Average)	Detection rates (%)
pMD-PRRSV-1	80	25	33.26	100
	40	25	34.31	100
	20	25	35.11	100
	10	25	36.21	100
	5	25	37.28	28
	2.5	25	No Ct	0
pMD-PRRSV-2	80	25	33.72	100
	40	25	34.83	100
	20	25	35.64	100
	10	25	36.81	100
	5	25	37.91	20
	2.5	25	No Ct	0

### Determination of test results

Based on the sensitivity analysis, interpretation criteria were established for the duplex fluorescence quantitative RT-qPCR assay targeting PRRSV-1 and PRRSV-2. The assay was deemed valid when both positive controls (HEX for PRRSV-1 and FAM for PRRSV-2) exhibited typical amplification curves, and the negative controls showed no amplification (Ct = 40 or undetermined). A sample was considered positive for PRRSV-1 if only the HEX channel showed a typical amplification curve with a Ct value ≤ 37.5, while the FAM channel showed no amplification (Ct = 40 or undetermined). Conversely, a sample was determined positive for PRRSV-2 if only the FAM channel showed a typical curve with a Ct value ≤ 39.5 and no amplification was observed in the HEX channel. If both channels exhibited amplification curves within their respective Ct thresholds (≤ 37.5 for HEX and ≤ 39.5 for FAM), the sample was identified as co-infected with PRRSV-1 and PRRSV-2. Samples with Ct values falling within the borderline range (≥ threshold and < 40) were classified as suspected positives and subjected to repeat testing with a doubled template volume. If the Ct value in the retest fell below the threshold, the sample was confirmed positive; otherwise, it was considered negative. Samples with no amplification in either channel (Ct = 40 or undetermined) were classified as negative for both PRRSV-1 and PRRSV-2.

### Repeatability analysis

The mixture of two standard plasmids (at a ratio of 1:1) with concentrations of 2×10^8^, 2×10^6^, 2×10^4^, and 2×10^2^ copies/μL (final reaction concentrations: 1×10^8^, 1×10^6^, 1×10^4^, and 1×10^2^ copies/μL) was used as a template for amplification to evaluate the repeatability of the assay. The results showed that the intra-assay and inter-assay CVs in this study were 0.22-1.07% and 0.52-1.28%, respectively (**Table 3**).

**Table 3:** The intra and inter assay results of the duplex RT-qPCR.

Standard plasmid	Concentration of template (copies/μL)	Intra-coefficient of variation		Inter-coefficient of variation	
		X ± SD	CV (%)	X ± SD	CV (%)
pMD-PRRSV-1	10^8^	12.34 ± 0.08	0.64	12.44 ± 0.11	0.99
	10^6^	19.08 ± 0.15	0.78	19.31 ± 0.21	1.09
	10^4^	25.63 ± 0.09	0.35	25.78 ± 0.33	1.28
	10^2^	32.58 ± 0.35	1.07	32.25 ± 0.24	0.74
pMD-PRRSV-2	10^8^	13.57 ± 0.11	0.81	13.43 ± 0.07	0.52
	10^6^	20.51 ± 0.09	0.43	20.11 ± 0.13	0.65
	10^4^	26.79 ± 0.06	0.22	26.79 ± 0.20	0.75
	10^2^	33.51 ± 0.21	0.62	32.98 ± 0.74	1.03

### Performance of the duplex RT-qPCR assay using clinical samples

A total of 356 clinical samples were detected using duplex RT-qPCR assay established in this study, and the positivity rates of PRRSV-1 and PRRSV-2 were 7.02% (25/356) and 30.34% (108/356), respectively. The co-infection rate of the samples was 1.69% (6/356). The 356 clinical samples were also detected using WOAH-recommended RT-qPCR and commercial universal PRRSV RT-qPCR detection kit. The positivity rates of PRRSV-1 and PRRSV-2 detected by WOAH-recommended RT-qPCR were 6.46% (23/356) and 28.37% (101/356), respectively, whereas those detected by commercial universal PRRSV RT-qPCR detection kit were 6.18% (22/356) and 28.93% (103/356), respectively (**Table 4**). The coincidence rates for detection of PRRSV-1 and PRRSV-2 between the assay established in this study and WOAH-recommended RT-qPCR were 99.44% and 98.03%, respectively, whereas those between the assay established in this study and commercial universal PRRSV RT-qPCR detection kit were 99.16% and 98.60%, respectively.

**Table 4:** Detection of PRRSV-1 and PRRSV-2 in clinical samples and comparison of three methods.

Method	PRRSV-1 Positivity Rate	PRRSV-2 Positivity Rate	PRRSV-1 Coincidence Rate	PRRSV-2 Coincidence Rate
The developed duplex RT-qPCR	25/356 (7.02%)	108/356 (30.34%)	–	–
WOAH-recommended RT-qPCR	23/356 (6.46%)	101/356 (28.37%)	99.44%	98.03%
Commercial kit	22/356 (6.18%)	103/356 (28.93%)	99.16%	98.60%

## Discussion

PRRS is one of the most common and economically important swine infectious diseases worldwide (Guo et al., 2018). A rapid and reliable method for PRRSV detection is essential for effective surveillance and control of the disease in pig herds. At present, a range of nucleic acid and antigen/antibody-based methods are available for the detection of PRRSV. Nucleic acid testing can be used for diagnostic purposes, and antibody testing can be used to assess PRRSV exposure or for herd serum monitoring. For example, ELISA is the best choice to evaluate the dynamic changes in antibody levels in vaccinated animals. In co-infection with PRRSV-1 and PRRSV-2, or PRRSV and other pathogens, qPCR-based methods demonstrate enhanced reliability and accuracy, particularly at low template concentrations. Currently, qPCR has become the most commonly used detection method for PRRSV.

To date, various qPCR detection methods for PRRSV, including TaqMan probe-based and SYBR Green-based assays, have been developed. Among these methods, some are used for the differential diagnosis of PRRSV-2 subtypes (Qiu et al., 2019), while others are designed for the simultaneous detection of PRRSV and other porcine pathogens (Zhao et al., 2019; Feng et al., 2024). At present, PRRSV-2 is the predominant epidemic strain in China, and most existing detection methods have been designed primarily to target PRRSV-2. However, due to the high incidence of PRRSV-2 mutation and recombination, the existing detection methods carry a risk of false negatives, highlighting the urgent need for further optimization and updating. Meanwhile, the detection rate of PRRSV-1 in China has been continuously increasing in recent years, with reports from more than 23 regions, and the pathogenicity of certain PRRSV-1 strains is also on the rise (Sun et al., 2023). Given that PRRSV-1 vaccines are not yet approved for use in China, the timely and accurate diagnosis of PRRSV-1 infections is particularly critical.

In this study, all full-length PRRSV-1 (n=74) and PRRSV-2 (n=512) genome sequences from China available in GenBank up to June 2024 were downloaded, and the consensus sequence was obtained by sequence alignment using MAFFT software. Subsequently, specific primers and probes targeting the conserved region of the ORF6 gene were designed for PRRSV-1 and PRRSV-2, respectively. Following optimization of the reaction conditions, a duplex RT-qPCR assay for the simultaneous detection of PRRSV-1 and PRRSV-2 was successfully established. The LOD was 8.42 copies for PRRSV-1 and 7.84 copies for PRRSV-2. No cross-reactivity was observed with other common swine pathogens, including PRV, PPV1, PCV2, PEDV, CSFV, SS, and GPS, indicating that the assay possesses excellent sensitivity and specificity. Moreover, repeatability tests demonstrated that the intra-assay and inter-assay CVs ranged from 0.22% to 1.07% and 0.52% to 1.28%, respectively, showing good reproducibility. Furthermore, we compared the established assay with the PRRSV detection method recommended by WOAH as well as a commercially available diagnostic kit, and the assay established in this study exhibited higher positivity rates. These findings suggest that the duplex real-time PCR assay developed in this study offers a higher detection rate and holds great potential for practical application.

In summary, we developed a duplex RT-qPCR assay capable of accurately diagnosing and differentiating PRRSV-1 and PRRSV-2 infections. The assay demonstrated excellent sensitivity, specificity, and reproducibility, providing a rapid and reliable tool for the timely detection and precise identification of PRRSV strains. Its application is expected to significantly improve PRRSV surveillance and facilitate more effective prevention and control efforts in the swine industry.

## Data Availability

The original contributions presented in the study are included in the article/supplementary material. Further inquiries can be directed to the corresponding authors.
